# Endocrine dysregulation in COVID-19: molecular mechanisms and insights

**DOI:** 10.3389/fendo.2024.1459724

**Published:** 2024-10-22

**Authors:** Cristiana Iosef, Andrei M. Matusa, Victor K. M. Han, Douglas D. Fraser

**Affiliations:** ^1^ Children’s Health Research Institute, London, ON, Canada; ^2^ Lawson Health Research Institute, London, ON, Canada; ^3^ Department of Pediatrics, Western University, London, ON, Canada

**Keywords:** endocrine, COVID-19, cortisol, adrenal glands, disease management

## Abstract

This review describes the impact of COVID-19 on the endocrine system, focusing on cortisol signaling and growth factor-induced endocrine resistance. As expected, SARS-CoV-2 infection induces systemic inflammation, resulting in stimulation of the adrenal glands leading to elevated cortisol levels with normal adrenocorticotropic hormone (ACTH) levels. The cytokine storm could also stimulate cortisol production. However, in some instances, cortisol levels rise independently of ACTH due to a phenomenon known as “pseudo-Cushing’s syndrome,” where adrenal glands become less responsive to ACTH. Plasma proteomic analyses showed that this pattern was variably observed among COVID-19 patients, potentially involving calcium dysregulation and GNAS-regulated activities, ultimately impacting the regulation of microvascular permeability. COVID-19 also exhibited a syndrome resembling endocrine resistance, governed by receptor tyrosine kinase signaling pathways. Mild cases displayed elevated activity of EGFR and MMP9, along with increased expression of survival factors like Bax and Bcl2. In contrast, more severe cases involved IGFR-I and enhanced NOTCH signaling, with altered expression of Bcl2, AKT1, and MAPK8. In summary, these findings describe the complex interplay between COVID-19 and endocrine pathology, particularly endocrine resistance. These insights suggest potential endocrine targets for therapeutic interventions to improve short- and long-term outcomes for COVID-19 patients.

## Endocrine profiles in COVID-19

To date, over 400,000 scientific reports on COVID-19 have been published worldwide and indexed in PubMed, with only a small percentage (~0.5%) addressing the impact of SARS-CoV-2 infection on the endocrine system. Severe COVID-19 is typically characterized by significant respiratory distress, low blood oxygen levels, the need for ventilation, and/or multi-organ dysfunction. Notably, obesity and diabetes have been identified as risk factors for severe infection since the early stages of the pandemic and have been extensively studied (1–4). Furthermore, it is increasingly acknowledged that patients with severe COVID-19 may experience adverse endocrine outcomes, including altered glucose metabolism, thyroid dysfunction, and adrenal insufficiency (1). This short review aims to outline the complex interactions between COVID-19 and the endocrine system disorders by synthesizing the current scientific knowledge obtained by targeted plasma proteomics and envisioning future research considerations. It is important to note that among the plethora of COVID-19 publications, only 0.08% of the cases refer to plasma proteomic profiles from different viewpoints, all excluding the endocrine complications. Our studies characterized the plasma proteomic profiles of patients with mild and severe COVID-19 by Olink targeted proteomics technologies exploring 3,072 proteins simultaneously (5, 6). We observed and predicted three categories of endocrine effects associated with the adrenocorticoid system: (i) calcium dysregulation that may lead to hormonal hypersecretion; (ii) novel actions of the guanine nucleotide binding protein (GNAS); and (iii) fluctuations in circulating growth factors.

SARS-CoV-2 has been detected in endocrine tissues (7), and the inflammatory processes that develop post-infection could directly or indirectly affect the endocrine tissues and their functions. Such observations have been made globally (8), with therapeutic strategies currently being developed to address the consequences (9). COVID-19 has been shown to affect various components of the endocrine system; recently, the hypothalamic-pituitary-adrenal (HPA) axis has gained interest along with the renin-angiotensin-aldosterone system (RAAS), and the thyroid system (1–4, 10). Disturbance of the RAAS due to viral infection can affect aldosterone and renin levels, leading to electrolyte disturbances and hypertension (11–13). Similarly, SARS-CoV-2 infection may lead to dysregulation of the HPA axis and alterations in cortisol levels. Some patients with severe COVID-19 may exhibit adrenal insufficiency or adrenal crisis, while others may have elevated cortisol levels due to the stress response and inflammation (1, 14, 15). Interestingly, the angiotensin-converting enzyme 2 (ACE2) receptor, which mediates SARS-CoV-2 viral entry into cells, is expressed in various tissues, including the adrenal glands and the pancreas, and has been implicated in the disturbance of the endocrine homeostasis. Additionally, SARS-CoV-2 infection can alter thyroid function, reflected by changes in the thyroid-stimulating hormone (TSH), free thyroxine (FT4), and free triiodothyronine (FT3) levels (16, 17). Some COVID-19 patients have also reported thyroid dysfunction, such as subacute thyroiditis or non-thyroidal illness syndrome (also known as ‘euthyroid sick syndrome’). Lastly, COVID-19 may also impact sex hormone levels, perhaps through changes in the functionality of the vascular supply to the primary sex organs (11, 18). In men, low testosterone levels have been linked to a more severe course of COVID-19, while in women, higher testosterone levels are associated with a stronger immune response (19).

COVID-19 impacts the insulin system by targeting, in part, the insulin-like growth factors (IGFs), as they utilize the same class of receptor tyrosine kinases (RTKs). In certain conditions, the RTKs can be shared by IGFs and insulin systems, and vice-versa. With regards to the insulin system, hyperglycemia, insulin resistance and new-onset diabetes are observed in some patients, particularly those who suffered severe COVID-19 (20–27). The exact mechanisms underlying these metabolic changes are not fully understood but may involve systemic inflammation, a stress response and the direct effects of the virus on the pancreatic beta cells (27, 28). COVID-19 may also affect the growth hormone/insulin-like growth factor (GH-IGF-1) axis and the gonadotropin-releasing hormone (GnRH) axis. Although we recently showed a comprehensive profile of the growth factors and their binding proteins based on the Olink plasma proteomics (5, 6), the changes in the IGFs system, the magnitude, and the significance of these alterations in COVID-19 patients require further investigation, especially with regards to the possible competition between IGF and insulin signaling mentioned above (20–22). The endocrine profile in COVID-19 is complex and can vary depending on factors such as the severity of illness, pre-existing hormonal disorders and individual patient characteristics (18).

**Figure 1:**
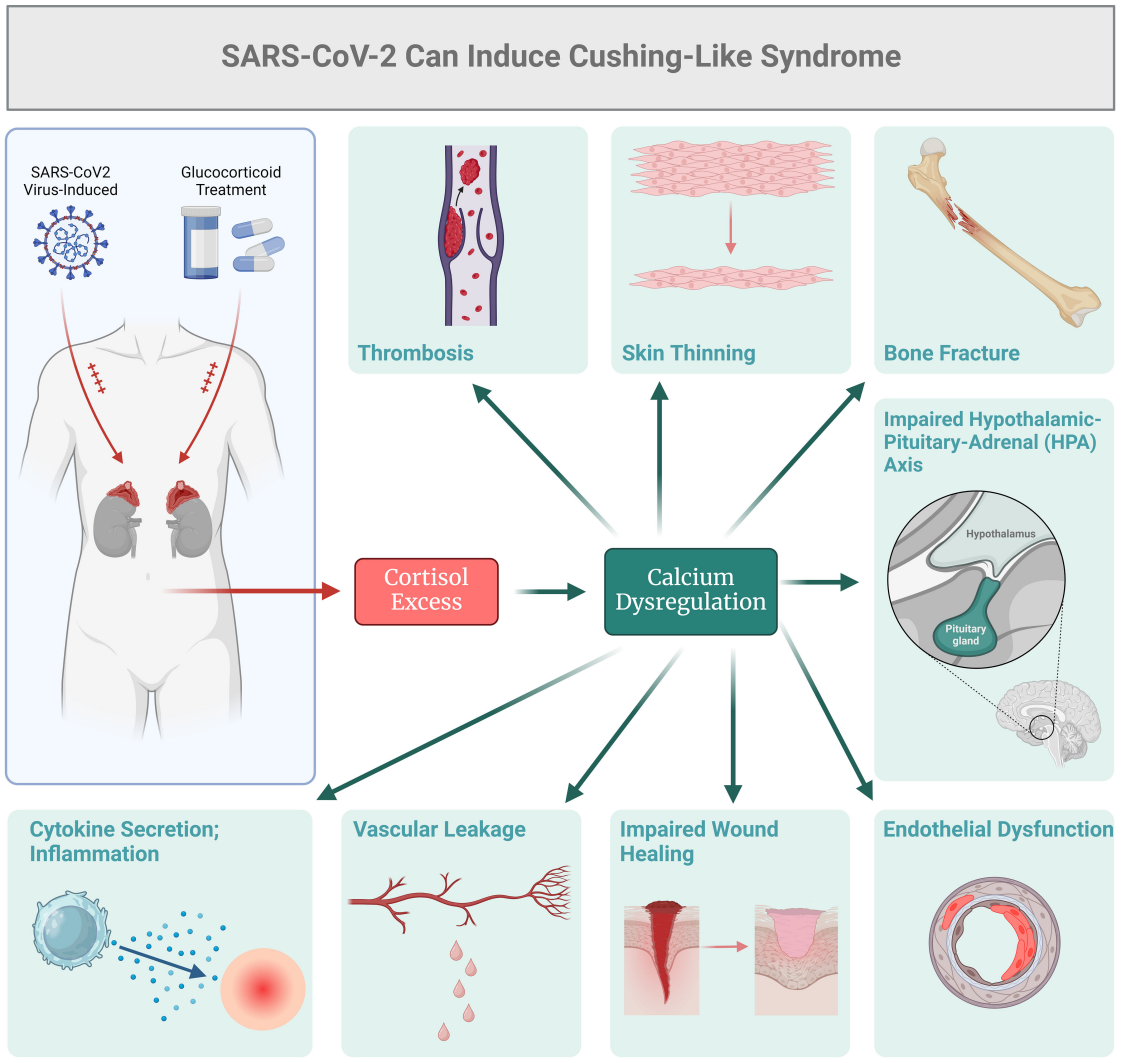
SARS-Cov2 can induce cortisol excess and Cushing's-like syndrome.

When investigating the endocrine status of current and recovered COVID-19 patients, reports suggest that some individuals may exhibit features resembling Cushing’s syndrome (29–31). As presented in **Figure 1**, one of the main causes of Cushing’s-like syndrome could be the use of exogenous glucocorticoids as a primary treatment for severe COVID-19. Patients with severe COVID-19 may receive glucocorticoids, such as Dexamethasone, to counteract systemic inflammation and the cytokine storm. In this context, treatment with glucocorticoids could lead to Cushing’s syndrome-like features, including hypertension, glucose intolerance, and muscle weakness (32–36). Shacham and Ishay (36) examined immune activation resulting from chronic endogenous glucocorticoid excess in Cushing's syndrome and explored how coronavirus infection might improve outcomes for COVID-19 patients treated with glucocorticoids. They concluded that a comprehensive understanding of the molecular and cellular mechanisms associated with both endogenous and exogenous glucocorticoids is crucial. This includes factors such as the timing of administration, dosage, duration of treatment, and specific formulations of these medications. While the exact mechanisms underlying these manifestations in COVID-19 are not yet known, pathology may vary, underscoring the potential for SARS-CoV-2 to affect adrenal function and cortisol metabolism. It is important to note that the Cushing’s syndrome-like manifestations observed in some COVID-19 patients may be transient and reversible, particularly in cases where they are associated with exogenous glucocorticoid administration or acute stress response (31–39). As glucocorticoid therapy is reduced with clinical improvement, these manifestations may improve or resolve. Some of the COVID-19 patients may still experience endogenous hypercortisolism as a result of viral infection, and thus the dysregulated cortisol production may persist. Again, this dysregulation can occur due to various factors, such as the stress-induced activation of the HPA axis, cytokine-mediated stimulation of adrenal cortisol synthesis, or direct effects of the virus on adrenal glands (31, 32). HPA axis can also be affected by systemic ionic dysbalance (39). Electrolyte disorders, such as potassium abnormalities, have been frequently reported as clinical manifestations of COVID-19. SARS-CoV-2 could affect potassium equilibrium via altered epithelial sodium channels (ENaC) activity (39). The incidence of hyperkalemia is due to the key role of furin which is hijacked by the virus, thus the decreased activity of ENaC would be expected, which causes retention of potassium ions and hyperkalemia (39). On the other hand, the envelope (E) protein of the SARS-CoV-2 virus forms cation-conducting channels in the endoplasmic reticulum Golgi intermediate compartment of infected cells (40). In this context, the calcium channel activity of E protein is associated with the inflammatory responses of COVID-19 (40).

Dysregulated calcium signaling pathway (KEGG: hsa04020) was notably significant across all COVID-19 datasets that we have analyzed using enrichment algorithms with Bonferroni correction (3–6). The three receptors involved in calcium signaling were: i) G Protein-Coupled Receptor (GPCR), which acts through Gs Alpha Subunit (GNAS) and affects parathyroid hormone receptor (PTHR) signaling, ii) Growth factors Receptor Tyrosine Kinase (RTK), which operates through Phospholipase C Gamma (PLCγ), Inositol 1,4,5-Trisphosphate Receptor (IP3R), and phosphatidylinositol (PI3), and iii) Voltage-Gated Calcium Channel (CaV1), which functions through Calmodulin and its associated kinases. These signaling pathways impact calcium homeostasis, cell proliferation, metabolism, tight junctions, and cell movement. One key mechanism that emerges from all our studies, and is relevant to endocrine function, involves the GNAS protein. Its role in cyclic adenosine monophosphate (cAMP) signaling is crucial for the regulation of calcium levels in the body in association with PTH.

## Plasma proteomics associated with SARS-CoV-2 infection may reflect an altered endocrine activity

In the past four years, our group has performed extensive plasma-targeted proteomics studies using the Olink technology on COVID-19 patients (3–6). The data suggests that, in addition to cortisol, growth factors, and other possible endocrinopathies, COVID-19 could potentially alter calcium homeostasis and the novel endocrine-related-proteins such as GNAS, which can potentially lead to further disruptions in calcium homeostasis and dysregulation of vascular permeability. All of this could be related to endocrine resistance due to SARS-CoV-2 induced complex pathology.

The GNAS gene, otherwise known for being involved in clinical phenotypes including pseudo-hypoparathyroidism (PHP) and pseudo-pseudo-hypoparathyroidism (PPHP), helps stimulate the activity of adenylate cyclase that controls the production of several hormones and regulates the activity of endocrine glands such as the thyroid, pituitary gland, ovaries and testes (gonads), and adrenal glands (41–44). GNAS-controlled activities that may be related to endocrine responses may affect, per se, hormone receptor signaling pathways, and hypothetically lead to endocrine resistance. For instance, GNAS signaling intersects with several pathways involved in hormone action, including cAMP/PKA and MAPK pathways, which are crucial for hormonal responses (41–44).

During viral infection, cellular calcium dynamics could be highly affected as dysregulation of host cell signaling cascades is elicited by SARS-CoV-2 (45). Calcium ions act as critical secondary messengers in cellular signaling pathways, including those involved in hormone receptor signaling and cell survival. Proper calcium levels are crucial for maintaining vascular integrity and permeability (45). Dysregulation of calcium homeostasis can lead to increased vascular permeability, which is associated with inflammatory processes, potentially leading to endocrine resistance (45). Moreover, alterations in calcium equilibrium and vascular permeability can disturb the microenvironment of hormone-sensitive cells, affecting their response to hormones in natural conditions and to hormone therapies. In addition, increased permeability may alter drug distribution and cellular signaling, potentially contributing to resistance mechanisms (45).

Vascular dysfunction in COVID-19 can also be associated with endocrine pathology that may lead to complications, including endothelial dysfunction, increased vascular permeability and thrombosis. An important example includes leaky blood vessels, which may impact hormone delivery and signaling in hormone-sensitive tissues, directly inducing inflammation with inflammatory cytokine release and direct effects on endothelial cells and other tissues (3–6).

The observed alterations in calcium channel function and GNAS-controlled activities could lead to disruptions in the regulation of vascular permeability, which may, in turn, induce endocrine resistance in the context of COVID-19. These disruptions affect intracellular signaling pathways crucial for hormonal responses and create an environment that hampers the effectiveness of endocrine therapies. Understanding these molecular and cellular changes can provide insights into endocrine resistance mechanisms in COVID-19 and inform potential therapeutic strategies to overcome resistance.

## Pathogenesis of Cushing’s-like syndrome in COVID-19

Conditions or factors that mimic Cushing’s syndrome without primary adrenal pathology are termed Cushing’s-like syndrome, and they may be observed in some COVID-19 patients. Again, precipitating factors may include direct viral infection, the cytokine storm and/or treatment with exogenous glucocorticoids. The GNAS-controlled activities, calcium balance, and vascular permeability could contribute to the clinical manifestations of Cushing’s-like syndrome, either directly or indirectly, through excessive cortisol or related hormonal imbalances (41–44). Calcium dysregulation can contribute to various symptoms seen in Cushing’s syndrome, such as muscle weakness and fatigue. On the other hand, GNAS mutations can also contribute to Cushing’s-like syndrome since they are associated with various endocrine disorders, including certain subtypes of Cushing’s syndrome, such as McCune-Albright syndrome (41–44). Dysregulated GNAS signaling can potentially lead to excessive production of cortisol or alterations in adrenal gland function, contributing to the development of this disease (41–44). Furthermore, calcium homeostatic effects and vascular permeability can be potentially observed in conditions associated with cortisol excess, including Cushing’s syndrome. This phenomenon acts as a feed-forward loop, where high cortisol levels can lead to alterations in calcium metabolism and induce changes in vascular permeability, thus resulting in multiple organ dysfunctions.

## Cortisol pathway activation in COVID-19: a comparative study between Western and Harvard University databases

**Figure 2:**
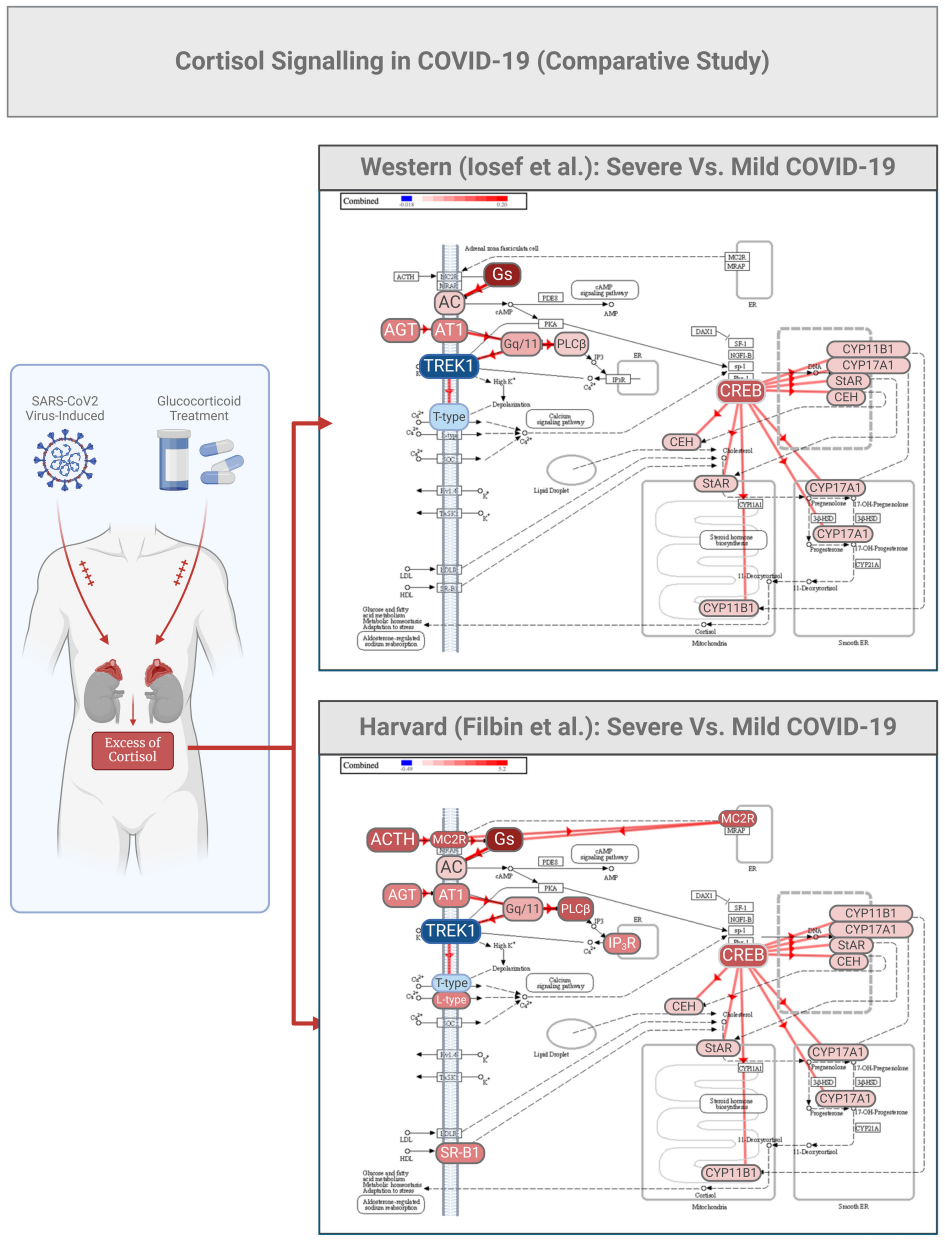
COVID-19 cortisol signaling in a comparative study between patient cohorts from Western and Harvard Universities. Partial data from the two studies (Ref 5, 39) were utilized for bioinformatic analysis to predict various aspects of cortisol signaling as initiated by potassium channels (TREK1). The two datasets were initially analyzed for differential expression using consistent thresholds for fold change (2-fold) and p-value (0.05). Subsequently, a meta-analysis was conducted using upset plot algorithms, enabling a non-directional cross-analysis that identified a pool of common proteins differentially expressed plasma proteins relevant to this study.

There are many studies describing genomics, epigenomics, transcriptomics, and proteomics profiles in COVID-19 (46–48), but very few have investigated the plasma proteome associated with this disease. Studies by our group, and that from Harvard Medical School (48), demonstrated similar changes in the cortisol signaling pathway in COVID-19 (5, 6) (**Figure 2**). Utilizing our own bioinformatics pipeline we compared our data with the repository published by Filbin et al.

The same comparative analysis also highlighted the activation of the angiotensin I (AGT1) 1/AT1 receptor system. Angiotensin I is usually cleaved by angiotensin-converting enzyme (ACE) to generate the active product angiotensin II, which is involved in maintaining blood pressure, body fluid and electrolyte homeostasis, as well as playing a role in the pathogenesis of essential hypertension and preeclampsia (48–50). In COVID-19, ACE acts as a pathological target, directly inducing smooth muscle cell vasoconstriction, influencing cardiac contractility and heart rate through the sympathetic nervous system, and altering kidney functions such as renal sodium and water absorption, concurrently stimulating the zona glomerulosa cells from the adrenal cortex to synthesize and secrete aldosterone (50). In **Figure 2**, we can further speculate that melanocortin receptors (MC2R) can be selectively activated by the adrenocorticotropic hormone ACTH in COVID-19, and TREK1 could activate phospholipase C (PLC beta), which catalyzes the formation of inositol 1,4,5-trisphosphate and diacylglycerol from phosphatidylinositol 4,5-bisphosphate. This latter enzymatic reaction requires calcium as a cofactor, and calcium plays a critical role in the intracellular transduction of many extracellular signals. PLC beta can be activated by two G-protein alpha subunits, thereby regulating the function of the endothelial barrier. Lastly, in the same **Figure 2**, it can be observed that the cortisol signaling pathway is predicted to exhibit high activation of the CREB/cytochrome P450 system (CYP).

Calcium regulation is critical for cellular homeostasis especially with infections such as SARS-CoV-2 (51, 52). When calcium channels open, they allow influx of calcium into the cells. Intracellular calcium levels modulate the inflammatory response, potentially enhancing or inhibiting the effects of dexamethasone (52). Calcium trafficking through the channels is vital for T cell activation (52). Calcium channels affect the release of cytokines, while dysregulation leads to unbalanced cytokine production and inflammation as seen in severe COVID-19 (50).

Furthermore, calcium ions themselves are secondary messengers for different signal transduction pathways especially when they can activate downstream signaling molecules such as calmodulin, calcineurin, and numerous kinases which in turn may regulate growth factor receptor (GF) signaling (53). Protein kinase C (PKC) and calcium/calmodulin-dependent protein kinase (CaMK), are two of the kinases that can phosphorylate GF receptors or their downstream effectors that were already targeted for COVID-19 therapy (53).

## Potential cortisol signaling-associated effectors driven by growth factors

**Figure 3:**
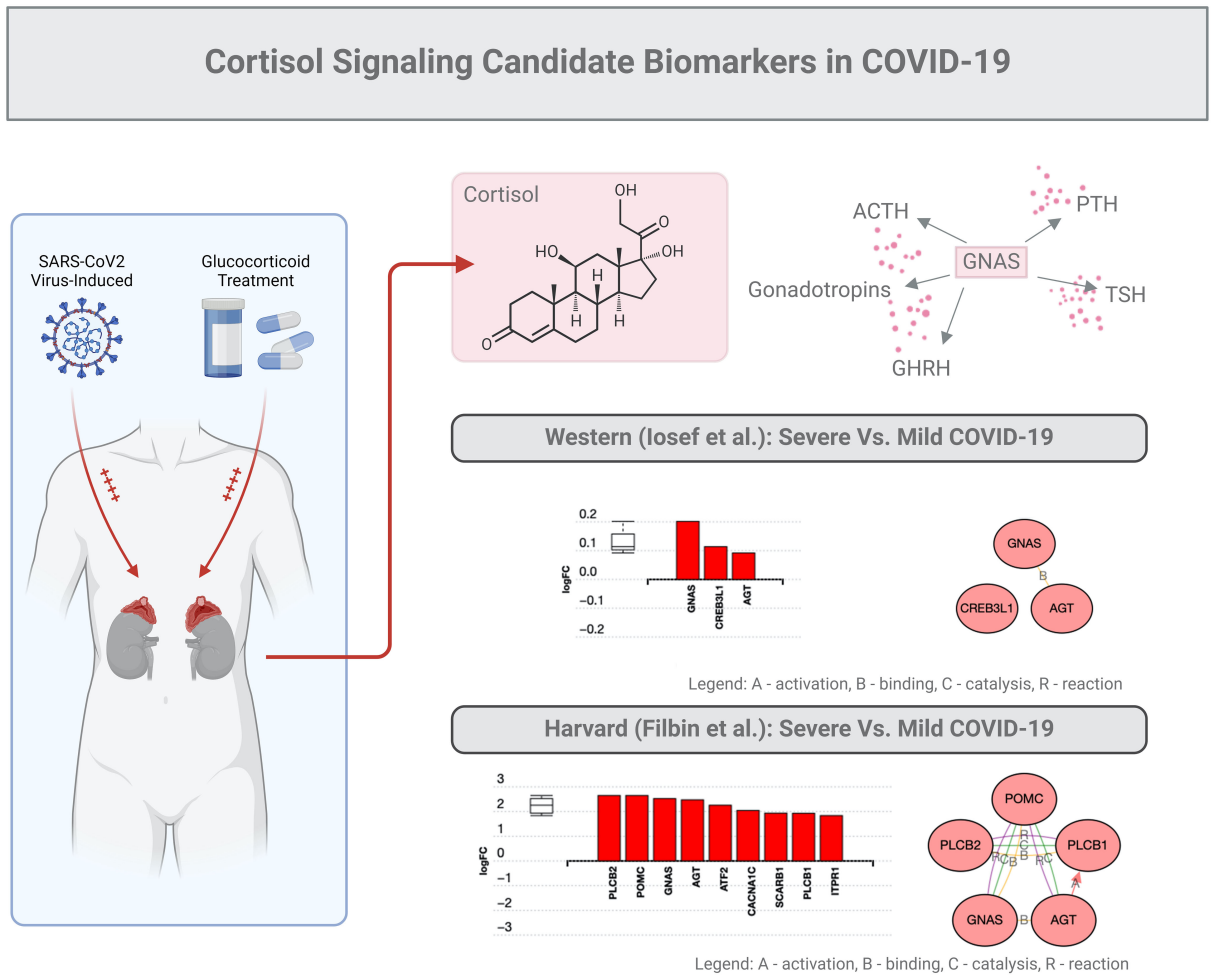
Cortisol signaling: candidate biomarkers in COVID-19. The same subset of data (201 proteins) described in the legend of **Figure 2** was used for an in-depth bioinformatic analysis focusing on the GNAS protein marker. This specific protein stimulates the activity of adenylate cyclase, an enzyme that plays a crucial role in regulating the production of several hormones that influence the function of endocrine glands, including the thyroid, pituitary gland, ovaries, testes, and adrenal glands.

In the study described above, GNAS was found to be a key factor upregulated almost 2-fold (**Figure 3**). This stimulatory G-protein alpha subunit peptide is an important element of the signal transduction pathway that links receptor-ligand interactions to the activation of adenylyl cyclase and a variety of cellular responses (41–43).

As mentioned above, GNAS is generally regarded as a ubiquitously expressed protein involved in several pathologies such as pseudo-hypoparathyroidism type 1a, fibrous dysplasia of bone, and even several pituitary tumors (41–43). GNAS functions downstream of several G protein receptors, including beta-adrenergic receptors, and alters the secretion of PTH, GHRH, ACTH, TSH, or gonadotrophins. In addition, GNAS, which regularly binds to angiotensin (AGT), can possibly interact with CREB molecules (41–43). Finally, we predict that GNAS may interact with Proopiomelanocortin (PMOC), a protein synthesized in corticotroph cells of the anterior pituitary associated with ACTH. In tissues, including the hypothalamus, placenta, and epithelium, these peptides have roles in pain and energy homeostasis, melanocyte stimulation, and immune modulation, especially inflammation. Overall, GNAS-related proteins could be proposed to interact with the cortisol signaling in COVID-19.

**Figure 4:**
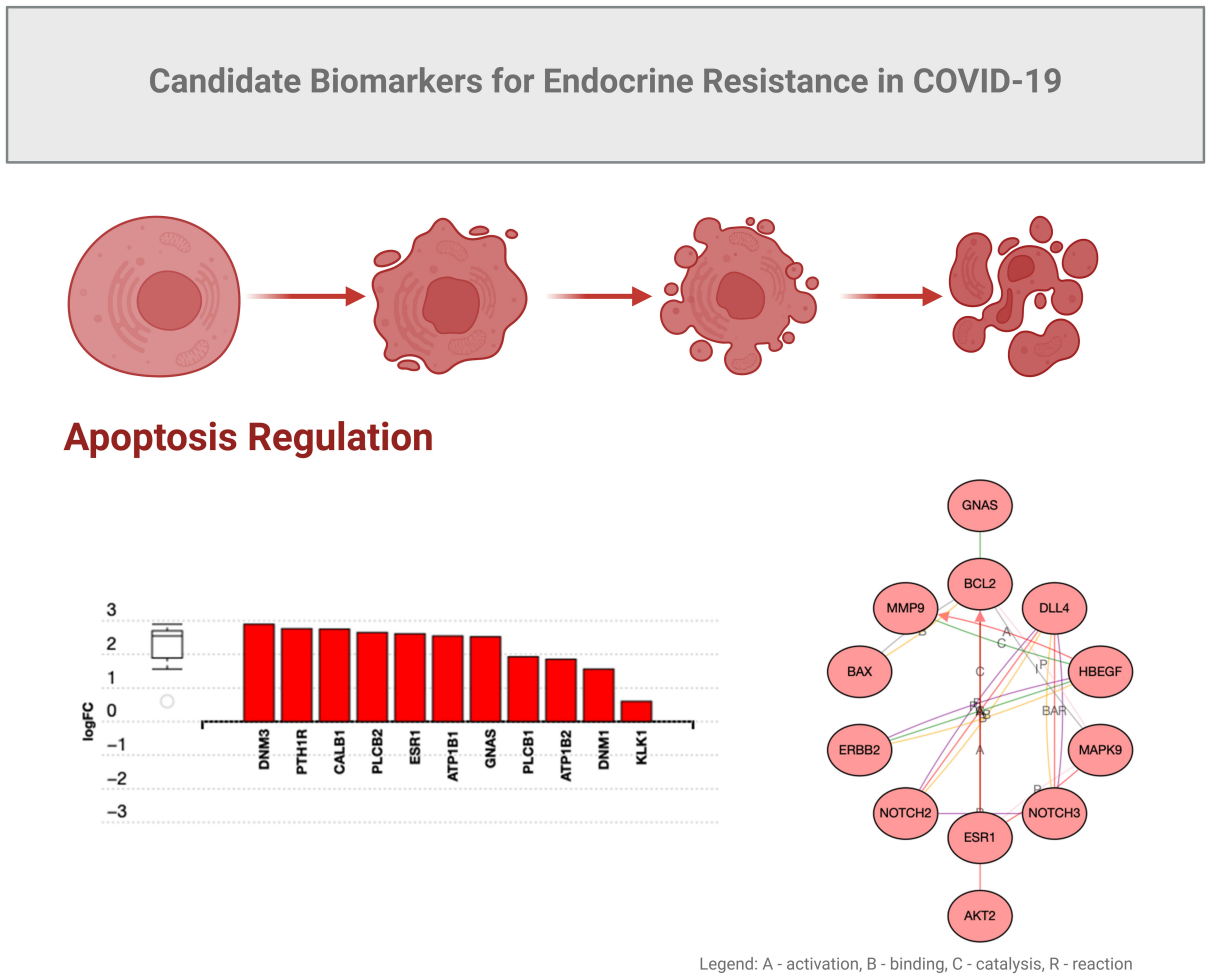
Candidate biomarkers for COVID-19 endocrine resistance.

**Figure 5:**
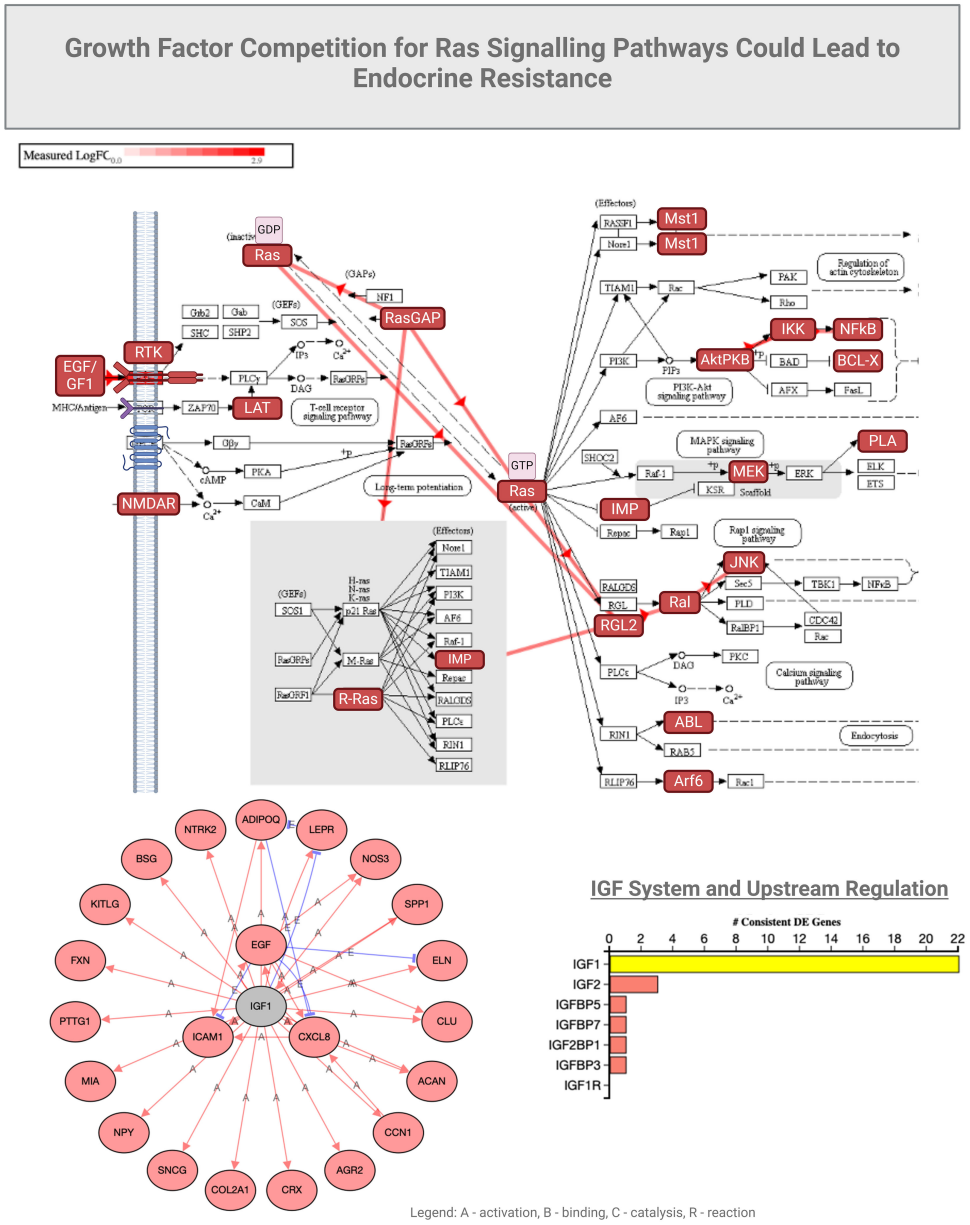
Growth factor competition for Ras signaling could lead to endocrine resistance.

GNAS is normally associated with RAS and KRAS activity in cancers (44). We observed that functions of GNAS could also be associated with programmed cell death in COVID-19 (**Figure 4**), possibly due to overlapping growth factor signals that may affect RAS signaling (**Figure 5**). In another study by Zhou S et al. (45), GNG7 (Guanine nucleotide-binding protein G) and GNAS proteins were also found to play “*a non-ignorable role in the progression of COVID-19*”. In this context, a consequence of overlapping signals can be Bax and Bcl2 dysregulation, which can also be part of the RTK signaling, ultimately regulating apoptosis under inflammatory conditions induced by SARS-CoV-2 (54, 55). Bax is a pro-apoptotic factor, while Bcl2 is anti-apoptotic, and the balance between these factors determines cell survival or apoptosis. We can speculate that in the context of COVID-19-associated *endocrine resistance*, elevated Bcl2 expression can help cells evade the apoptotic signals that would typically result from hormone therapy, thus contributing to *resistance*. It is clear that COVID-19 can disrupt various hormonal pathways through inflammatory responses and direct viral effects on endocrine organs, even creating local memory (1, 55). However, many effects could be a consequence of the anti-COVID-19 therapy (53). As such, endocrine disruptions would lead directly to hormonal resistance.

As we elaborated on RAS signaling we observed that stress and inflammation caused by COVID-19 can even induce a state of amplified cellular survival, proliferation and migration signaling, as evidenced also by the elevated EGFR, MMP9, and other survival factors. These changes can mirror the mechanisms underlying endocrine resistance by creating an environment where cells become less responsive to hormonal regulation and more reliant on alternative survival pathways. This environment can mimic the conditions that lead to endocrine resistance, where hormone-sensitive cells become less responsive to hormonal signals due to the activation of alternative survival and growth pathways. This observation can lead to understanding the broader implications of COVID-19 on endocrine health and resistance to endocrine therapies.

## Alterations of growth factor signaling in mild COVID-19

In mild COVID-19 cases (hospitalized for oxygen therapy only), we observed elevated activity of epithelial growth factor receptor (EGFR) and metalloproteinase (MMP9), along with increased expression of survival factors like Bax and Bcl2, which can be related to endocrine resistance through several mechanisms. Endocrine resistance, *per se*, refers to the reduced response of hormone-sensitive cells to endocrine therapies, often seen in, but not exclusive to, conditions such as certain cancers. EGFR is a receptor tyrosine kinase (RTK) that, when activated, initiates a cascade of downstream signaling pathways supporting cell proliferation, survival, and differentiation (56). The impact of EGFR on endocrine signaling is manifested by chronic activation of EGFR, which can alter the normal signaling balance within cells and potentially lead to reduced sensitivity to hormonal signals. As EGFR activation can stimulate pathways (e.g., MAPK, PI3K/Akt) that overlap or interfere with hormone receptor pathways, it thereby promotes resistance to hormone-based therapies (56). The EGF-EGFR axis may also have a role in tissue remodeling and inflammation, affecting metalloproteinase-9 (MMP9), an enzyme involved in the breakdown of extracellular matrix components (57, 58), which could potentially influence hormone receptors. Elevated MMP9 may lead to increased degradation of extracellular matrix components and growth factor receptors, and alter the cellular microenvironment that affects hormone receptor expression and function, contributing to endocrine resistance. Moreover, MMP9 could release growth factors sequestered in the matrix, further activating signaling pathways like EGFR and exacerbating the resistance mechanisms. Through MMP9 activities and aberrant release of growth factors, these molecules can cause signaling competition by overlapping induction of RTKs. It is important to note that future research should investigate the role of EGFR in COVID-19 management, particularly as clinical trials are already exploring the repurposing of EGFR inhibitors for treatment. For example, nimotuzumab, an anti-cancer monoclonal antibody targeting EGFR, has been repurposed for COVID-19 and studied for its potential to modulate the immune response and reduce inflammation. Nimotuzumab may help mitigate severe inflammatory responses in COVID-19 patients, potentially improving clinical outcomes (59). However, research remains limited, and more comprehensive clinical trials are needed to establish its efficacy and safety specifically for COVID-19 treatment.

## Endocrine profile in severe COVID-19

Unlike in mild COVID-19, the severe cases of COVID-19 that were admitted to intensive care units (3–6) and showed the involvement of insulin-like growth factor receptor I (IGF-IR), enhanced NOTCH signaling, along with altered expression levels of Bcl2, AKT1, and MAPK8 that can lead to endocrine resistance through various intricate mechanisms (**Figure 5**). IGF-IR is a receptor that, when activated by insulin-like growth factors (IGFs), triggers downstream signaling pathways that promote cell growth, survival, and metabolism; these factors have been studied in COVID-19 in association with clinical parameters (60–63). Enhanced IGF-IR signaling may lead to increased cellular survival and proliferation, similar to EGFR activation. The result is a reduced efficacy of hormone-based therapies due to the activation of alternative survival pathways (e.g., PI3K/Akt and MAPK pathways), which can interfere with, or bypass, hormone receptor signaling.

Our studies also indicated that NOTCH signaling can be crucial in cell differentiation, proliferation, and apoptosis. Enhanced NOTCH signaling could potentially lead to changes in cell fate and survival. Increased NOTCH signaling would also contribute to endocrine resistance by making cells less responsive to apoptotic signals induced by hormone therapies. In addition, NOTCH signaling can interact with other pathways, such as AKT and MAPK, to further complicate the cellular response to hormonal treatments (64).

Similar to the EGFR state observed in mild COVID-19, we also detected altered expression of Bcl2, AKT1, and MAPK8. Again, increased levels of Bcl2 promote cell survival by inhibiting apoptosis, making cells resistant to treatments that rely on inducing cell death. AKT1, part of the PI3K/Akt pathway, is crucial for cell survival, growth, and metabolism in COVID-19 (65). Increased AKT1 activity can enhance cell survival, resistance to apoptosis, and contributes to endocrine resistance. In addition, MAPK8 (JNK, Stress-Activated Protein Kinase) is usually involved in stress responses and can influence apoptosis and cell proliferation. Altered MAPK8 signaling would not only affect the cellular response to stress and apoptosis, but also contribute to resistance mechanisms. It is thus predicted that endocrine disruptions leading to resistance may involve increased activation of IGFR-I and NOTCH pathways, along with altered levels of Bcl2, AKT1, and MAPK8 creating a cellular environment that promotes survival and proliferation (64, 66). Such an environment can reduce the efficacy of hormone therapies by promoting alternative pathways that support cell survival, independent of the presence of endocrine treatments.

In summary, the activation of IGFR-I and enhanced NOTCH signaling, coupled with altered expression of Bcl2, AKT1, and MAPK8 in severe COVID-19 may lead to an environment that favors cell survival and proliferation. These observed changes could lead to endocrine resistance by promoting pathways that bypass or interfere with hormone receptor signaling, making cells less responsive to hormone therapies. Understanding these interactions can provide insights into how acute COVID-19 influences endocrine health and resistance mechanisms, and provide clinical guidance towards more effective treatment strategies.

## Methodological limitations

There are very few studies available for comparison, with the notable exception of the research conducted by Filbin et al. (39) at Harvard University, which makes our data sets unique. Both Filbin et al. and Iosef et al. (5) analyzed patients at Day 0/1, 3, and 7 post-admission with severe COVID-19, all of whom presented with severe disease characterized by bilateral pneumonia. For our cross-comparison aimed at identifying pathways related to endocrine dysfunction, we utilized data from Day 3 for both studies.

In Filbin et al.’s study, there were 109 patients with severe COVID-19 and 78 COVID-negative controls, while Iosef et al. examined 22 patients with severe COVID-19 and 22 healthy controls. A notable distinction between the two studies lies in the technology employed: Filbin et al. used the Olink Explore 1536 platform, whereas Iosef et al. utilized the Olink Explore 3072, which allows for the analysis of a greater number of differentially expressed proteins.

We acknowledge that confounding factors and publication biases may influence our results; however, our primary aim was to conduct a meta-analysis using algorithms that facilitate a comprehensive comparison of data sets derived from Olink technology, specifically analyzing plasma samples from patients with severe COVID-19. We maintained consistent thresholds for p-values and fold change (FC) in our analysis. While the patient numbers varied, recruitment was age- and sex-matched in both studies, and the criteria for severe COVID-19 were similar.

After aligning and meta-analyzing the data sets, we identified 1,780 differentially expressed proteins in our dataset and 783 in the Harvard dataset. The difference in numbers arises from the distinct panels of markers used for targeted proteomics in each study. Notably, we found that 201 markers were common between the two studies, forming the basis for our comparative analysis. Clinical parameters, including age, sex, comorbidities (such as hypertension, COPD, cancer, and chronic kidney disease), baseline medication, and laboratory parameters (like blood counts and X-ray findings) were also comparable across the studies, including confirmation of sepsis and administered intervention drugs.

## Conclusions

This review examines the endocrine implications of COVID-19, focusing on cortisol signaling and growth factor-induced endocrine resistance, highlighting the impact of SARS-CoV-2 on the endocrine system. COVID-19 often causes elevated cortisol levels with normal ACTH levels due to systemic inflammation, illness-induced stress, and cytokine storms. Sometimes, cortisol levels rise independently of ACTH, resembling “pseudo-Cushing’s syndrome.” Plasma proteomics suggests this variation may involve calcium dysregulation and GNAS-controlled activities, affecting vascular permeability. COVID-19 also presents endocrine resistance syndromes through activation of receptor tyrosine kinase pathways. Mild cases show elevated EGFR and MMP9 activity, while severe cases involve IGF-1R and enhanced NOTCH signaling, altering Bcl2, AKT1, and MAPK8 expression. The medical conclusion is that COVID-19 potentially impacts the endocrine system, particularly described here, through altered cortisol signaling and endocrine resistance mechanisms. These changes include elevated cortisol levels with normal ACTH levels, resembling “pseudo-Cushing’s syndrome,” and variations in receptor tyrosine kinase pathways, with different patterns in mild and severe cases. These endocrine alterations should be considered when developing targeted therapeutic interventions to improve patient outcomes. Developing interventions aimed at preventing severe cases could be a crucial direction for future research and clinical practice.
